# Supplementary material for: ATM and CDK2 control chromatin remodeler CSB to inhibit RIF1 in DSB repair pathway choice
Source: Nat Commun. 2017 Dec 4;8:1921. doi: 10.1038/s41467-017-02114-x (PMC5715124; doi:10.1038/s41467-017-02114-x)
Supplement: Supplementary file 1 — Supplementary Information [file 41467_2017_2114_MOESM1_ESM.pdf]

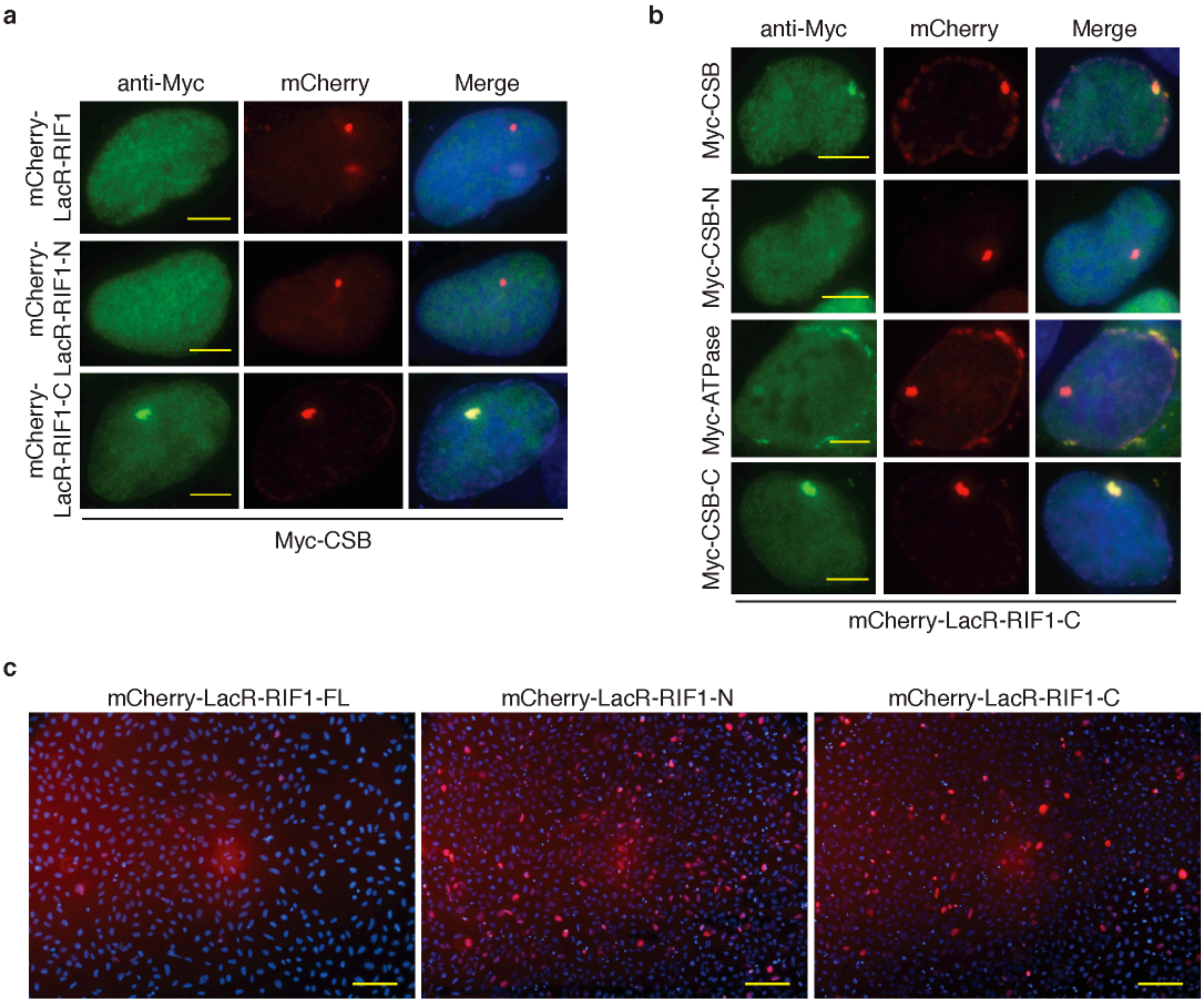

Supplementary Figure 1. RIF1-C interacts with CSB-C. (**a**) Immunofluorescence of U2OS-265 cells that were co-transfected with Myc-CSB and various mCherry-LacR-RIF1 alleles as indicated. Cell nuclei were stained with DAPI in blue in this and subsequent figures. Scale bars, 5 μm. (**b**) Immunofluorescence of U2OS-265 cells that were co-transfected with mCherry-LacR-RIF1-C and various Myc-tagged CSB alleles as indicated. Scale bars, 5 μm. (**c**) Immunofluorescence of expression of mCherry-LacR-RIF1 alelles in U2OS-265 cells from (a). Scale bars, 50 μm.

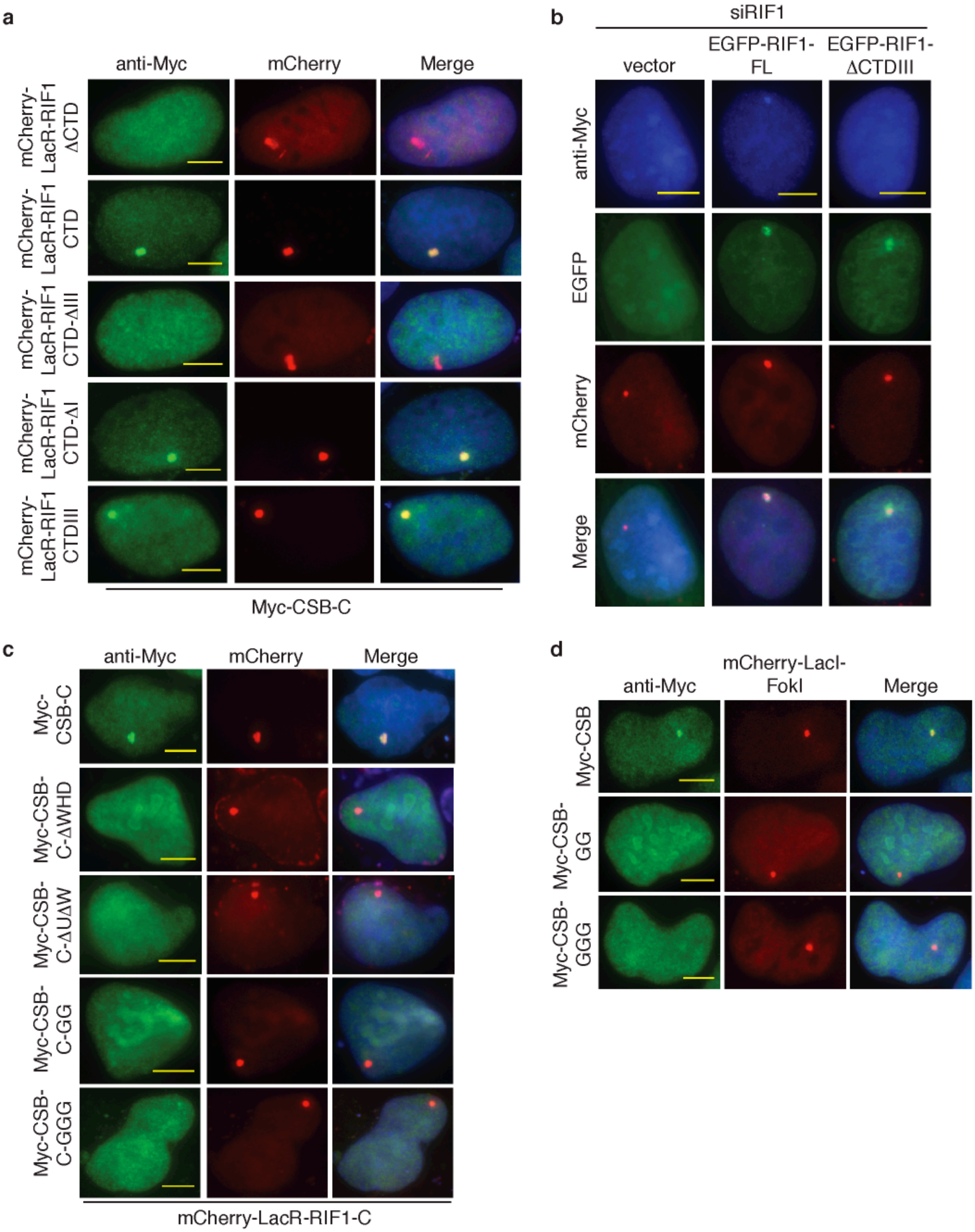
a
anti-Myc
mCherry
Merge
mCherry-LacR-RIF1 ΔCTD
mCherry-LacR-RIF1 CTD
mCherry-LacR-RIF1 CTD-ΔIII
mCherry-LacR-RIF1 CTD-ΔI
mCherry-LacR-RIF1 CTDIII
Myc-CSB-C
b
siRIF1
vector
EGFP-RIF1-FL
EGFP-RIF1-ΔCTDIII
anti-Myc
EGFP
mCherry
Merge
c
anti-Myc
mCherry
Merge
Myc-CSB-C
Myc-CSB-C-ΔWHD
Myc-CSB-C-ΔUΔW
Myc-CSB-C-GG
Myc-CSB-C-GGG
mCherry-LacR-RIF1-C
d
anti-Myc
mCherry-LacI-FokI
Merge
Myc-CSB
Myc-CSB-GG
Myc-CSB-GGG

Supplementary Figure 2. CSB interacts with RIF1 and is recruited by the CTD of RIF1 to sites of DSBs. (**a**) Immunofluorescence of U2OS-265 cells that were co-transfected with Myc-CSB-C and various mCherry-LacR-RIF1 alleles as indicated. Scale bars, 5 μm. (**b**) Immunofluorescence. 24 hr post transfection with siControl and siRIF1, U2OS-265 cells were transfected with the vector alone (EGFP), EGFP-RIF1-FL or EGFP-RIF1-ΔCTDIII and then induced for FokI expression 48 hr post transfection. Fixed cells were immunostained with anti-Myc antibody (in blue). Scale bars, 5 μm. (**c**) Immunofluorescence of U2OS-265 cells that were co-transfected with mCherry-LacR-RIF1-C and various Myc-tagged CSB-C alleles as indicated. Scale bars, 5 μm. (**d**) Immunofluorescence of U2OS-265 cells expressing various Myc-tagged CSB alleles as indicated. 48 hr post transfection, cells were induced for FokI expression and fixed 6 h post FokI induction. Scale bars, 5 μm.

a

```
                                                                     *                *  *
CSB   1417  ALLPTTEHDDLLVEMRNFIAFQAHTDGQASTREILQEFESKL----SASQSCVFRELLRNLCTFHRTSGG-EGIWKLKPEYC
            -----HHHHHHHHHHHHHHHHHH--------HHHHHHHH----    ---HHHHHHHHHHHHHHEE------ -EEEEE-----
RAP74 445   GPLGSGDVQVTEDAVRRYLTRK-----PMTTKDLLKKFQTKKTGLSSEQTVNVLAQILKRLNPERKMIND-KMHFSLKE
RAP30 175     RARADKQHVLDMLFSAFEKHQ----YYNLKDLVDI---------TKQPVVYLKEILKEIGVQNVKGIH-KNTWELKPEY
ELL2  200       TISQRPYRDRVIHLLALKA-----YKKPELLARLQKDGVN-QKDKNSLGAILQQVANLNSK------DLSYTLKDYVF
ELL   200     SGVSQR-PFRDRVLHLLALR-----PYRKAELLLRLQKDGL-TQADKDALDGLLQQVANMSAK------DGTCTLQDCMY
TAF1  797   KRANTHIRDFLQVFIYRLFWKSKDRPRRIRMEDIKKAF--------PSHSESSIRKRLKLCADFKRTGMD-SNWWVLKSDF
CAF1  519   KQKAMITDPMDLLRLFDGV-----QDSTFSLGTVTEIAQKNL----PQYNKQTIKNTIKEYAIRSSGKGDLPRKWVIKDAQN
```

b

```
                                                                 *               *  *
Human       ALLPTTEHDDLLVEMRNFIAFQAHTDGQASTREILQEFESKLSASQSCVFRELLRNLCTFHRTSGGEGIWKLKPEYC
Chimp       ALLPTTEHDDLLVEMRNFIAFQAHTDGQASTREILQEFESKLSASQSCVFRELLRNLCTFHRTSGGEGIWKLKPEYC
Dog         PPPPTTEHDDLLVEMRNFIAFQARVDGQASTQEILQEFESKLSASQSCVFRQLLRNLCTFHRTSSGEGIWKLKPEYC
Rat         APPCSTEHDALLVDMRNFIAFQAQVDGQASTQEILQAFESKLSVAQSCVFRELLRNLCNFHRTPGGEGIWKLKPEYC
Chicken     PAPGSTEYDELLVDVRNFIAFQARVDGEASTQEILQEFESKLPAAQSCVFRELLRNLCSFHRNPNGEGVWRLKPEFR
Snake       VPPTATEHDELLLDIRNFIAFQAHVPGQASTQEILHEFESKLTMEQSCVFRELLRNLCTFHRRSNGEGLWKLKPEFT
Frog        PSAGTTEHDELLADMRNFIAFQARVDGEASTQELLQEFENKLSQKQSCVFRELLRRLCTFHRNTDGTGVWRLKPEFH
Fish        PPAPPTEHDELLVDLRNFVAFQASVDGEATTQEVLEYFKPRLTQKQAPVFRELLRSICDFHRTCGQEGIWRLKENFR
Spider      TSKPATQHDELLVDIRNFIAFRAAVDGQATTKEIVEAFRDKLPIQQNAVFKSLLSKICDFSRSSDGDGIWHLKEEFR
Octopus     DDVASAVDIEFISDIRNFIAFQCNINGEATTEELIKEFKPKIPVSDSAKFKAMLKEICNFNRKN-GIGFWRLKQDFR
Cress       GSSSRVGSLQPEVLIRKICSFVQQKGGSADTTSIVNHFRDIVSFNDKQLFKNLLKEIATLEKDQN-RSFWVLKSEYK
Algae       GGGATRADAEKILD-DICRFLRDRPGGAAPTGLIVDAFGHAV--RDKGLFRRLLKQAARLEKGA-GTAQWVLRDHFA
Yeast       ARSKEINSNTKTLENIRAYLQKQNNFFSSSVSILNSIGVSLSDKEDVIKVRALLKTIAQFDKE---RKGWVLDEEFR
```

c

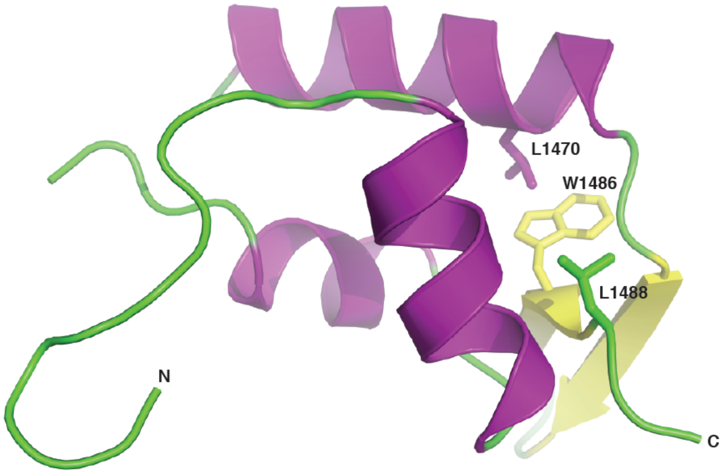

Supplementary Figure 3. CSB contains a winged helix domain (WHD). (**a**) Sequence alignment of the CSB WHD with other WHD found in structural homologs. PDB codes are RAP74: 1I27; RAP30: 1BBY; ELL2: 2EFN; ELL: 2DOA; TAF1: 4RGW; CAF1: 5EJO. Secondary structure prediction of helices (H) and β-strands (E) of CSB WHD as generated by the server JPRED4 (www.compbio.dundee.ac.uk/jpred4)

is indicated underneath the sequence of CSB. Identical amino acids to those found in CSB are colored in grey; similar amino acids (charged RHKDE, polar uncharged STNQY, hydrophobic AVILMFWCPG) are colored in cyan. For CSB, the sequence is colored according to whether a match is made to an amino acid of a structural homolog in the order of preference: identical>similar>no match. Asterisks indicate the amino acids in CSB that were mutated in this study. (**b**) Sequence alignment of the WHD of CSB homologs from human to yeast. Identical amino acids in homologs to those found in CSB are colored in grey; similar amino acids as in (a) are colored in cyan. For CSB, sequence coloring is as in (a). Accession numbers are: NP_000115.1 (*Homo sapiens*); XP_009438634 (*Pan troglodytes*); XP_534944 (*Canis lupus familiaris*); NP_001100766 (*Rattus norvegicus*); XP_421656 (*Gallus gallus*); XP_007442212 (*Python bivittatus*); OCA28283 (*Xenopus tropicalis*); XP_005815483 (*Xiphophorus maculatus*); KFM67945 (*Stegodyphus mimosarum*); XP_014774958 (*Octopus bimaculoides*); NP_179466 (*Arabidopsis thaliana*); XP_002502040 (*Micromonas commoda*); AJR54981 (*Saccharomyces cervisae* YJM689). (**c**) Cartoon representation of three-dimensional structure of the WHD of human CSB based on the C-terminal domain of the RAP74 subunit of the human transcription factor IIF (PDB 1I27) as generated from FFAS[36] alignment. Helices are shown in purple; β strands are in yellow; random coils are in green. Three amino acids that were mutated in this study are shown in stick representation. The figure was generated using PyMOL (www.pymol.org).

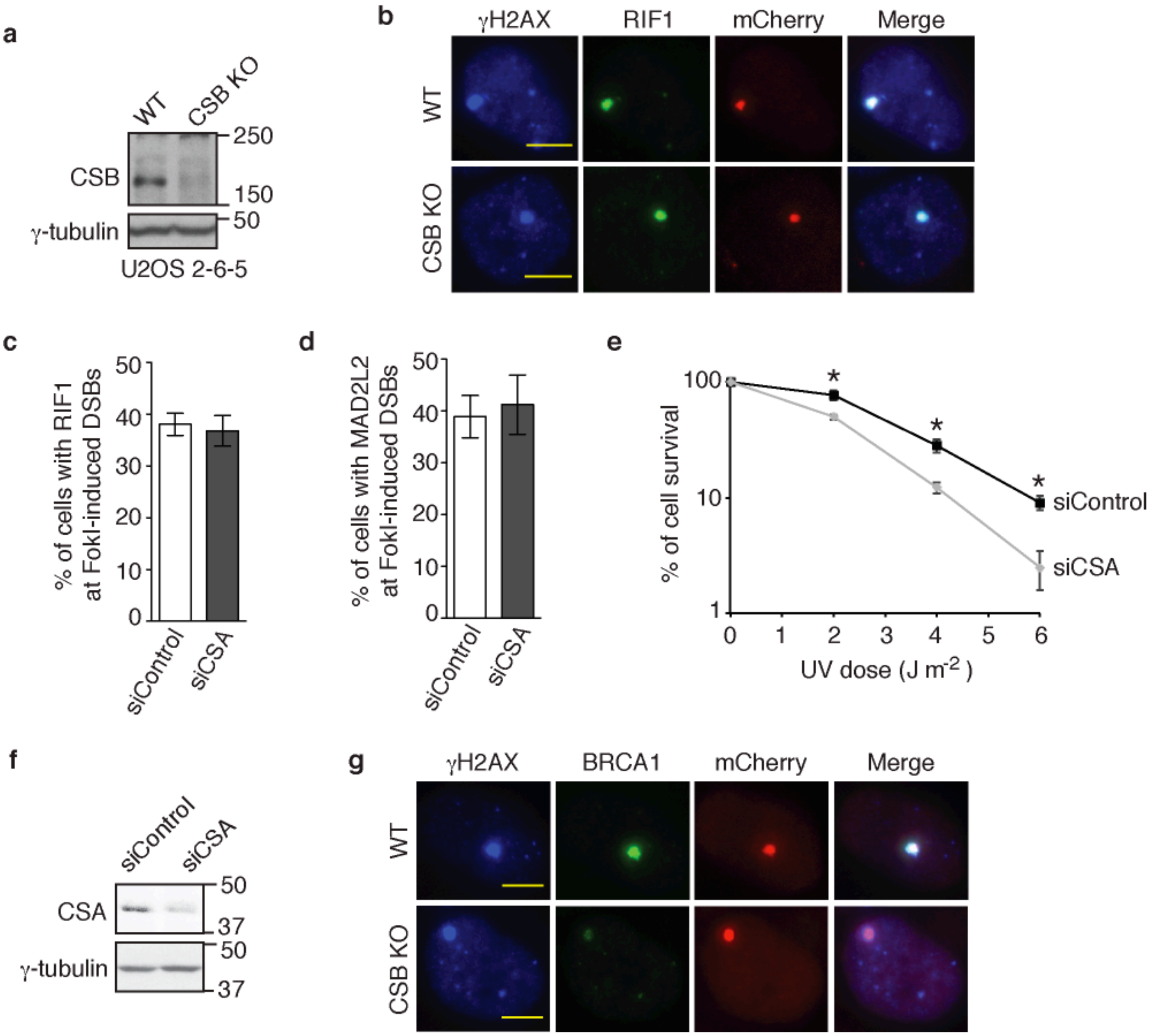

Supplementary Figure 4. CSB but not CSA restricts the RIF1-MAD2L2 pathway. (**a**) Western analysis of wild type (WT) and CSB knockout U2OS-265 cells. The γ-tubulin blot was used as a loading control in this and subsequent figures. (**b**) Immunofluorescence of U2OS-265 parental and CSB KO cells. Cells were fixed 4 h post FokI induction and co-stained with anti-RIF1 and anti-γH2AX antibodies. γH2AX staining was used to mark the FokI-induced damage site. Scale bars, 5 μm. (**c**) Quantification of the percentage of siControl- and siCSA-expressing U2OS-265 cells with RIF1 accumulated at FokI-induced DSBs. At least 500 cells were scored for each independent experiment in a blind manner. SDs from three

independent experiments are indicated. (**d**) Quantification of the percentage of siControl- and siCSA-expressing U2OS-265 cells with MAD2L2 accumulated at FokI-induced DSBs. Scoring was done as described in 4c. SDs from three independent experiments are indicated. (**e**) Clonogenic survival assays of siControl- and siCSA-expressing U2OS cells following treatment with UV irradiation. Standard deviations from three independent experiments are indicated. *$P<0.05$ (Student *t* test). (**f**) Western analysis of U2OS cells transfected with scambled siRNA (siControl) or siRNA against CSA. Immunoblotting was done with anti-CSA and anti-γ-tubulin antibodies. (**g**) Immunofluorescence of U2OS-265 parental and CSB KO cells. Cells were fixed 4 h post FokI induction and co-stained with anti-BRCA1 and anti-γH2AX antibodies. γH2AX staining was used to mark the FokI-induced damage site. Scale bars, 5 μm.

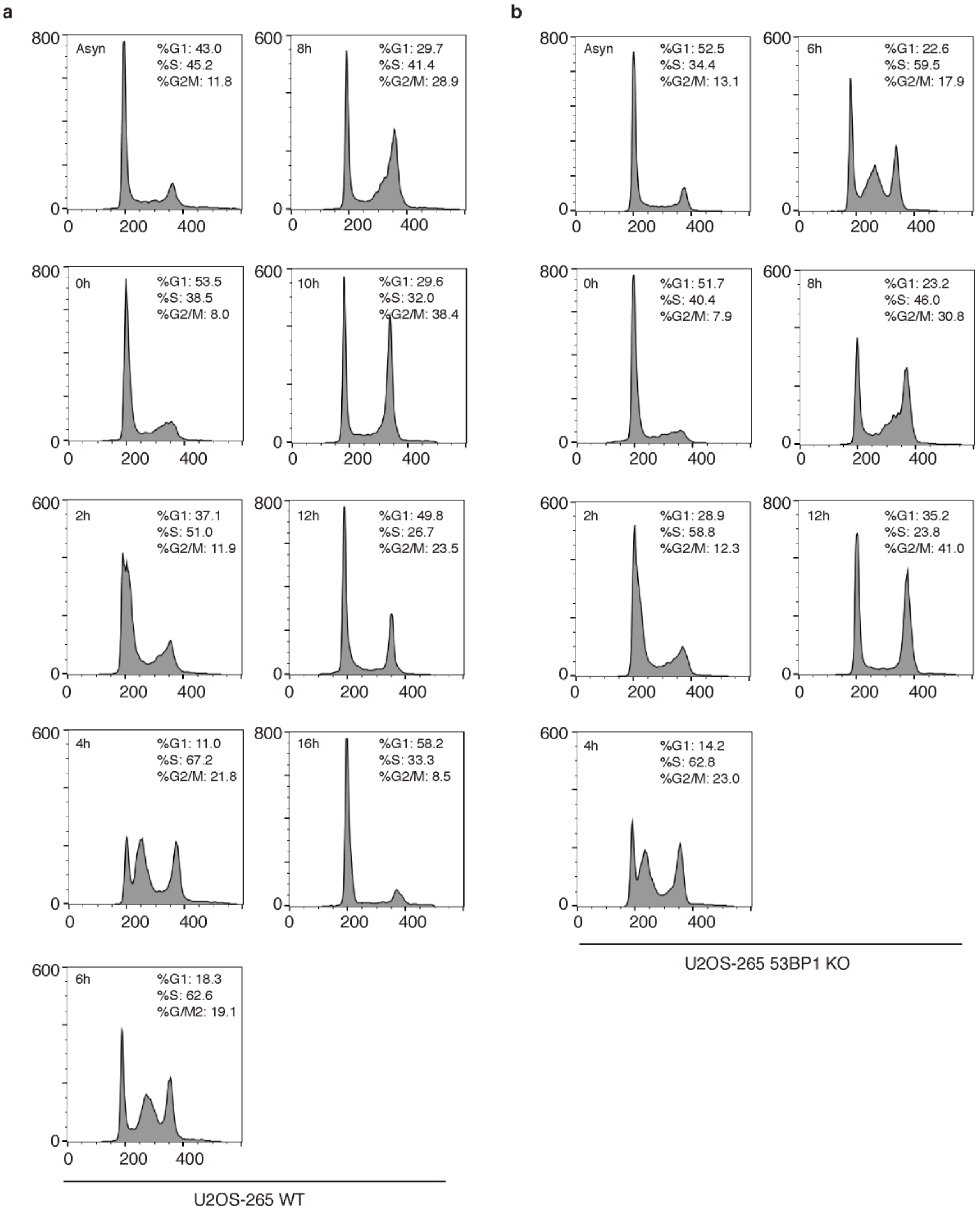

Supplementary Figure 5. Loss of 53BP1 has little effect on cell cycle progression. (**a**) FACS analysis of synchronized parental U2OS-265 cells. y axis, cell number; x axis, relative DNA content on the basis of

staining with propidium iodide; 0-16 h, cells were released for 0-16 h from a double thymidine block; Asyn, asynchronous population. (**b**) FACS analysis of synchronized U2OS-265 53BP1 KO cells. y axis, cell number; x axis, relative DNA content on the basis of staining with propidium iodide; 0-12 h, cells were released for 0-12 h from a double thymidine block; Asyn, asynchronous population.

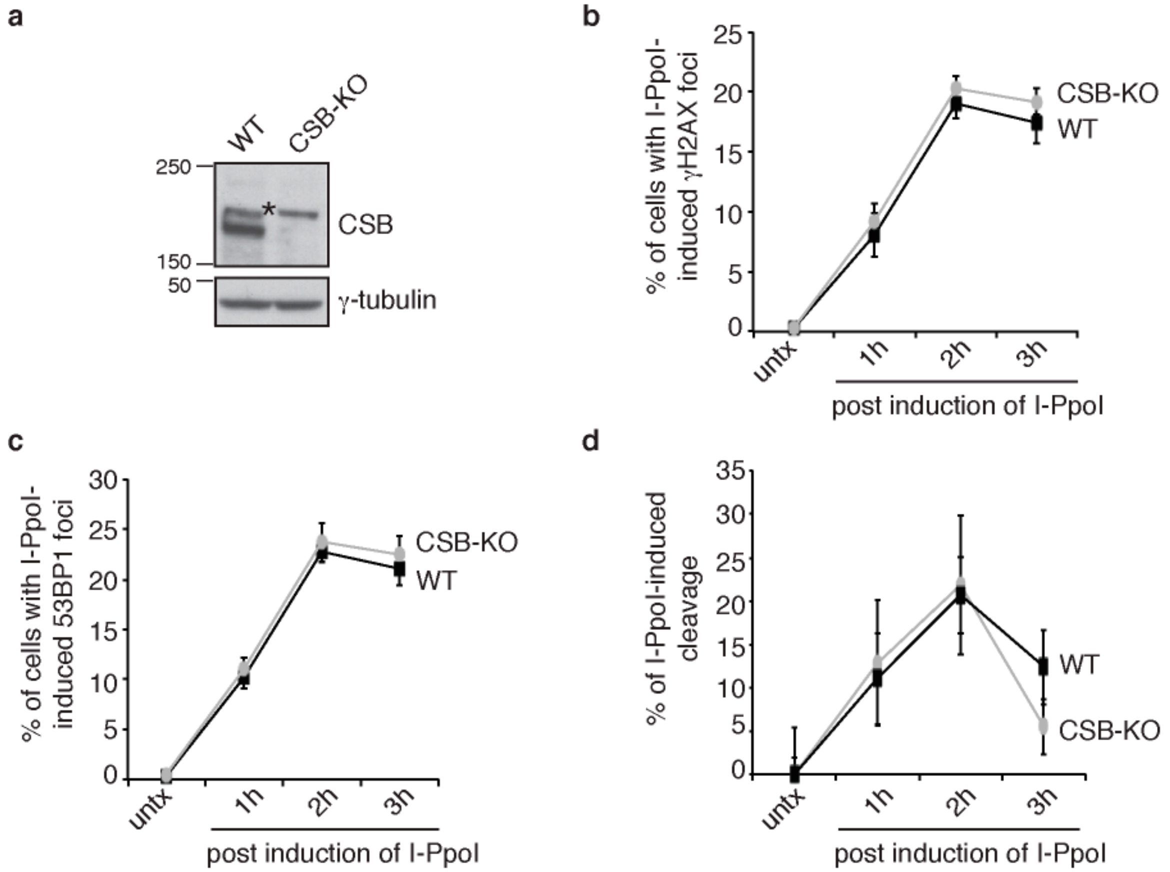

Supplementary Figure 6. Knockout of CSB does not affect the production of I-PpoI-induced DNA cleavage. (**a**) Western analysis of hTERT-RPE WT and CSB KO cells. The asterisk indicates the non-specific band. (**b**) Quantification of the percentage of hTERT-RPE parental (WT) and CSB KO cells exhibiting I-PpoI-induced γH2AX foci. A minimum of 500 cells were scored for each independent experiment in a blind manner. SDs from three independent experiments are indicated. (**c**) Quantification of the percentage of hTERT-RPE parental (WT) and CSB KO cells exhibiting I-PpoI-induced 53BP1 foci. Scoring was done as in 6b. SDs from three independent experiments are indicated. (**d**) Quantification of the percentage of I-PpoI-induced DNA cleavage from hTERT-RPE WT and CSB KO cells on chromosome 1. SDs from three independent experiments are indicated.

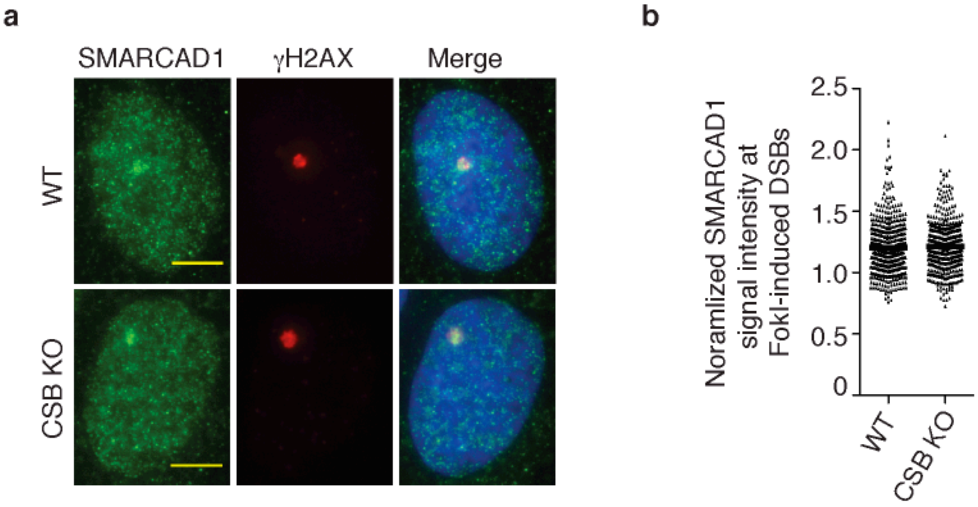

Supplementary Figure 7. Loss of CSB does not affect SMARCAD1 recruitment to sites of FokI-induced DSBs. (**a**) Immunofluorescence of U2OS-265 WT and CSB KO cells. Cells were fixed 6 h post FokI induction and co-stained with anti-SMARCAD1 and anti-γH2AX antibodies. γH2AX staining was used to mark the FokI-induced damage site. Scale bars, 5 μm. (**b**) Quantification of the intensity of SMARCAD1 signal at the site of FokI-induced DSBs from 7a. Cells positive for γH2AX were used for analysis of SMARCAD1 signal intensity. The respective numbers of cells analyzed for WT and CSB KO were 469 and 470.

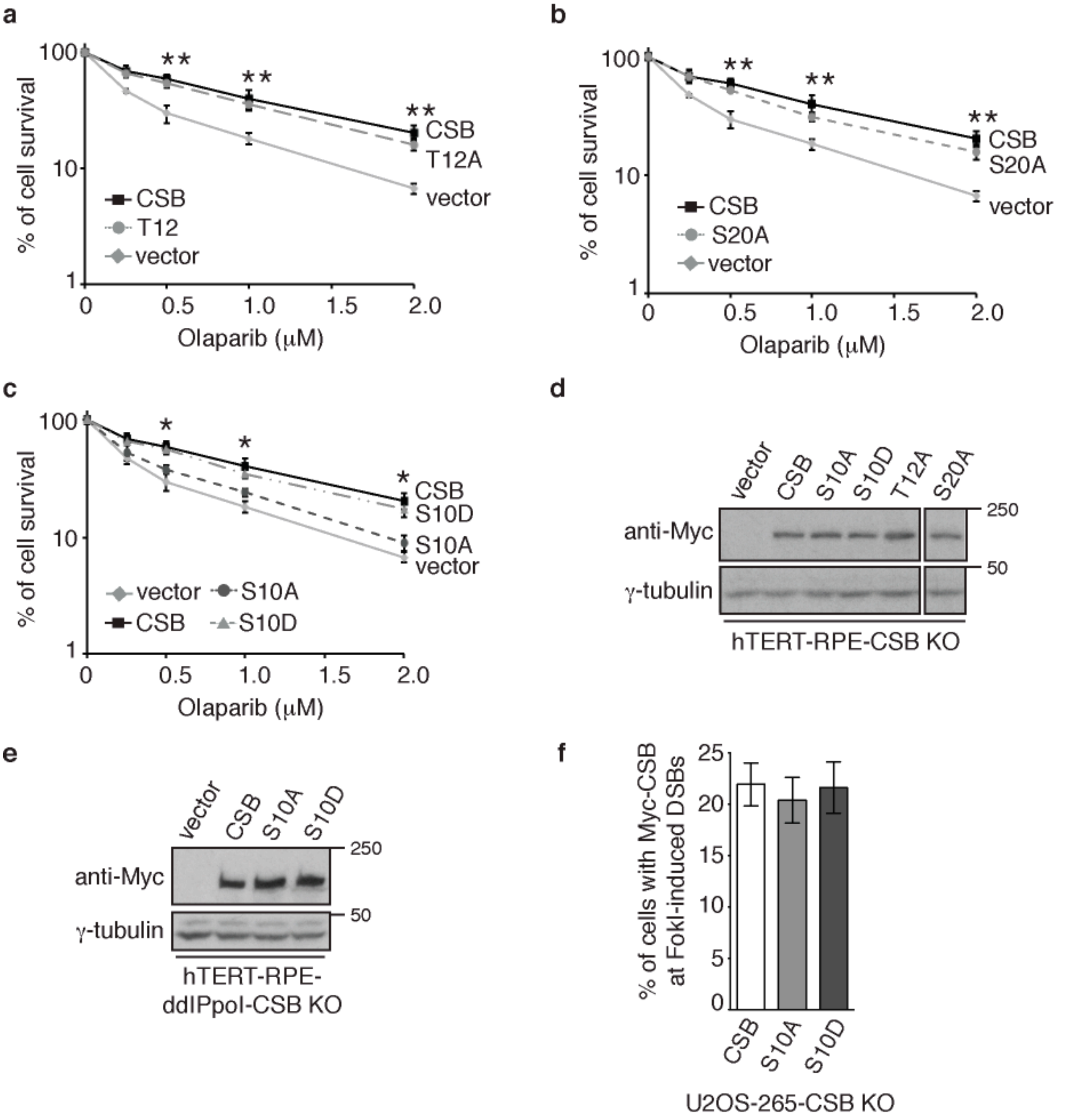

Supplementary Figure 8. CSB phosphorylation on S10 is necessary to support cell survival in response to the PARP inhibitor olaparib. (**a-c**) Clonogenic survival assays of olaparib-treated hTERT-RPE CSB-KO cells complemented with the vector alone or various Myc-tagged CSB alleles as indicated. Standard deviations from three independent experiments are indicated. ***P*>0.05 (Student *t* test) for comparison between CSB and T12A (a), between CSB and S20A (b). **P*<0.05 (Student *t* test) for comparison

between CSB and S10A (c). (**d**) Western analysis of hTERT-RPE CSB-KO cells stably expressing the vector alone or various Myc-tagged CSB alleles as indicated. (**e**) Western analysis of ddI-PpoI-expressing hTERT-RPE CSB-KO cells stably expressing the vector alone or various Myc-tagged CSB alleles as indicated. (**f**) Quantification of the percentage of Myc-CSB, Myc-CSB-S10A and Myc-CSB-S10D-expressing U2OS-265 cells exhibiting anti-Myc staining at FokI-induced DSBs. A total of 250 cells positive for anti-Myc staining were scored for each independent experiment in a blind manner. SDs from three independent experiments are indicated.

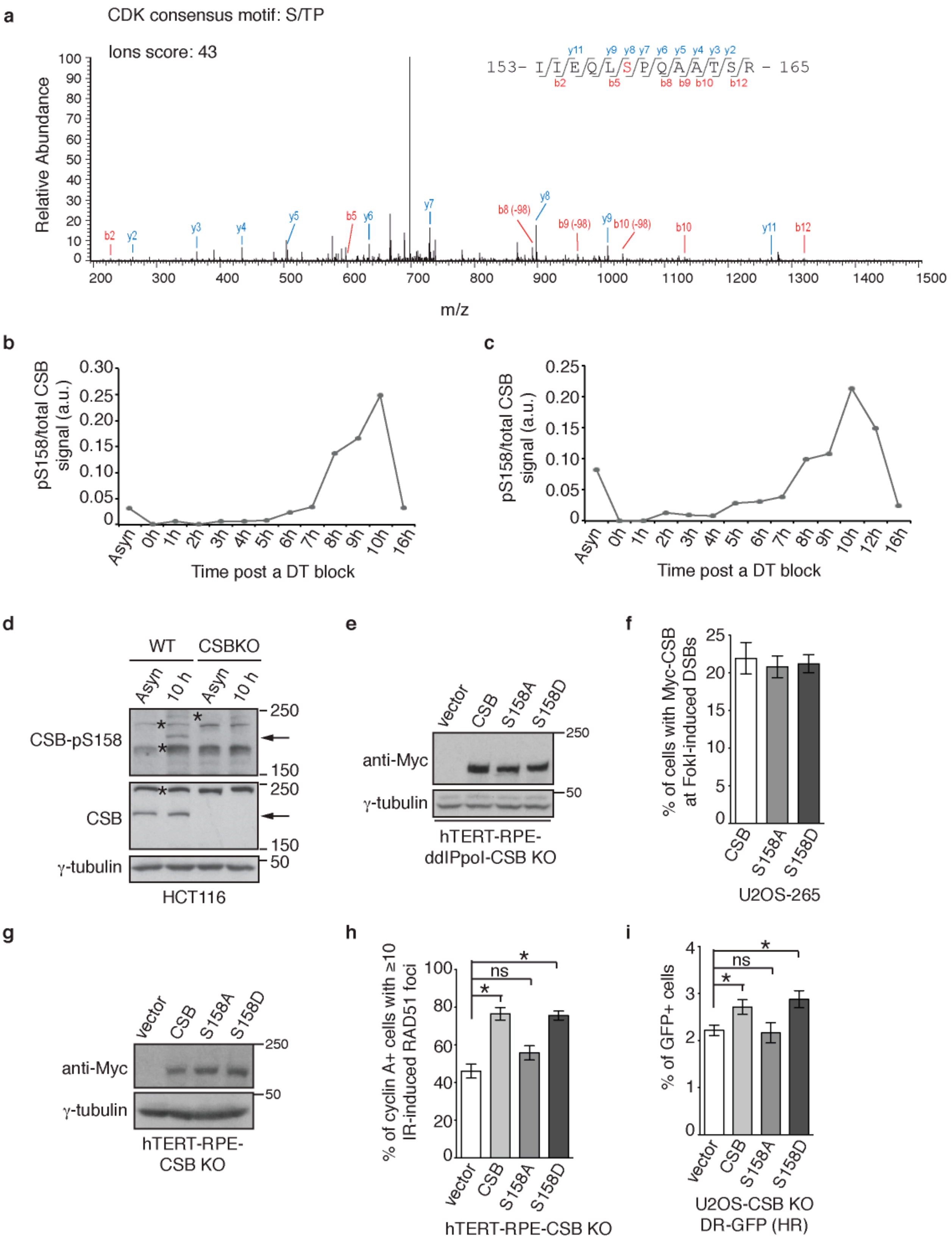
a
CDK consensus motif: S/TP
Ions score: 43
153- I/I/E/Q/L/S/P/Q/A/A/T/S/R - 165
Relative Abundance
m/z
b
pS158/total CSB signal (a.u.)
Time post a DT block
c
pS158/total CSB signal (a.u.)
Time post a DT block
d
WT
CSBKO
Asyn
10 h
CSB-pS158
CSB
γ-tubulin
HCT116
e
vector
CSB
S158A
S158D
anti-Myc
γ-tubulin
hTERT-RPE-ddIPpol-CSB KO
f
% of cells with Myc-CSB at FokI-induced DSBs
U2OS-265
g
anti-Myc
γ-tubulin
hTERT-RPE-CSB KO
h
% of cyclin A+ cells with ≥10 IR-induced RAD51 foci
hTERT-RPE-CSB KO
i
% of GFP+ cells
U2OS-CSB KO DR-GFP (HR)

Supplementary Figure 9. CSB phosphorylation on S158 is necessary for HR-mediated repair of DSBs. (**a**) Fragmentation spectrum of a trypic peptide surrounding pS158 (indicated in red) of CSB identified by MS/MS analysis of immunoprecipitated and phospho-enriched Flag-CSB. The peptide shown was identified with a Mascot ions score of 43 and an expect score of 0.0044. (**b**) Quantification of the CSB-pS158 signal from the western shown in Fig. 8b. Quantification was done with ImageJ. (**c**) Quantification of the CSB-pS158 signal from a second western using an independently prepared synchronzied cell lysate. Quantification was done with ImageJ. (**d**) Western analysis of HCT116 WT and CSB KO cells that were either asynchronous (Asyn) or 10 h post release from a double thymidine block. The arrow indicates the position of CSB-pS158. Asterisks indicate non-specific bands. (**e**) Western analysis of ddI-PpoI-expressing hTERT-RPE CSB-KO cells complemented with the vector alone, various Myc-tagged CSB alleles. (**f**) Quantification of the percentage of Myc-CSB, Myc-CSB-S158A and Myc-CSB-S158D-expressing U2OS-265 cells exhibiting anti-Myc staining at FokI-induced DSBs. A total of 250 cells positive for anti-Myc staining were scored for each independent experiment in a blind manner. SDs from three independent experiments are indicated. (**g**) Western analysis of hTERT-RPE CSB-KO cells stably expressing the vector alone or various Myc-tagged CSB alleles as indicated. (**h**) Quantification of the percentage of cyclin A+ cells with 10 or more IR-induced RAD51 foci. hTERT-RPE CSB KO cells stably expressing the vector alone or various Myc-tagged CSB alleles as indicated were treated with 2 Gy IR and fixed 4 hr post IR. A minimum of 500 cells were scored for each independent experiment in a blind manner. SDs from three independent experiments are indicated. *$P<0.05$. ns: $P>0.05$ (Student *t* test). (**i**) HR-mediated repair of I-SceI-induced DNA DSBs. SDs from three independent experiments are indicated. *$P<0.05$. ns: $P>0.05$ (Student *t* test).

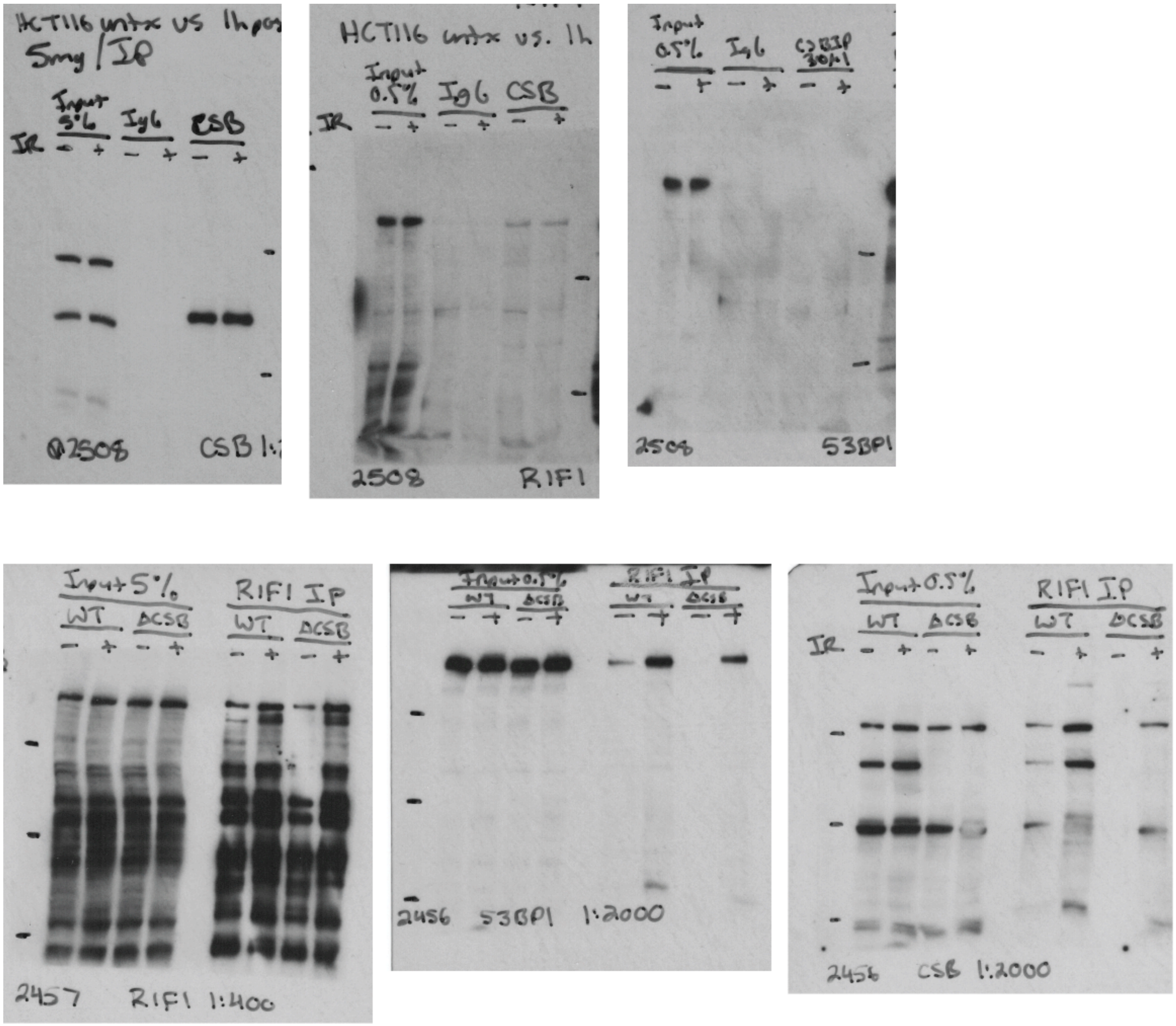

Supplementary Figure 10. Source data for western blots shown in Fig. 1h (top panel) and 1i (bottom panel).

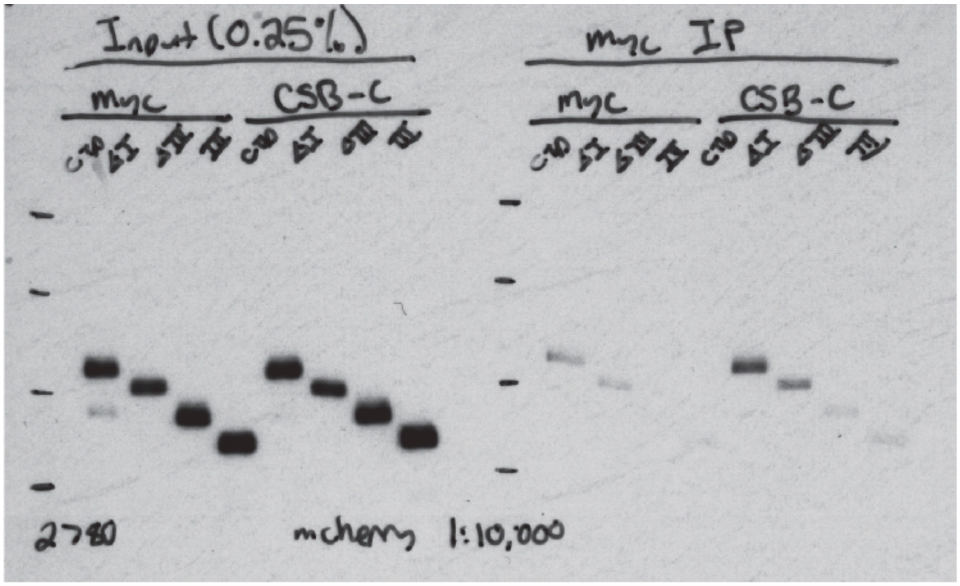

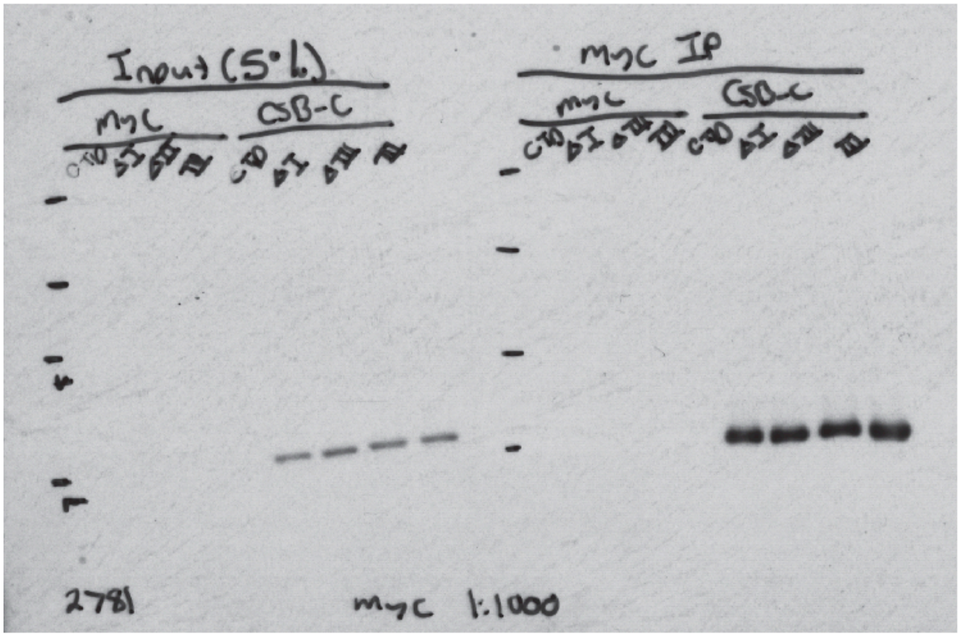

Supplementary Figure 10. Source data for western blots shown in Fig. 2g.

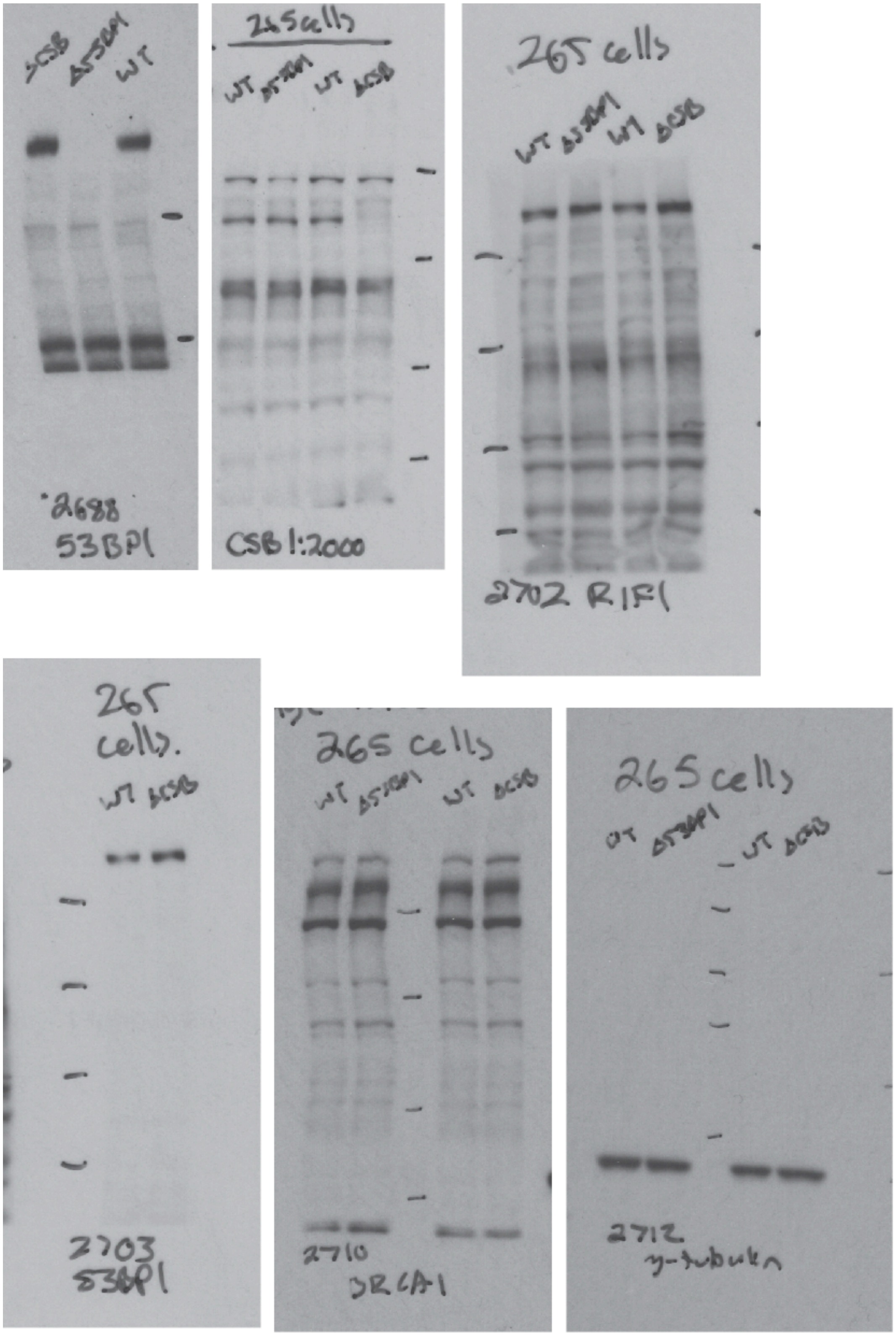

Supplementary Figure 10. Source data for western blots shown in Fig. 4c and 4j.

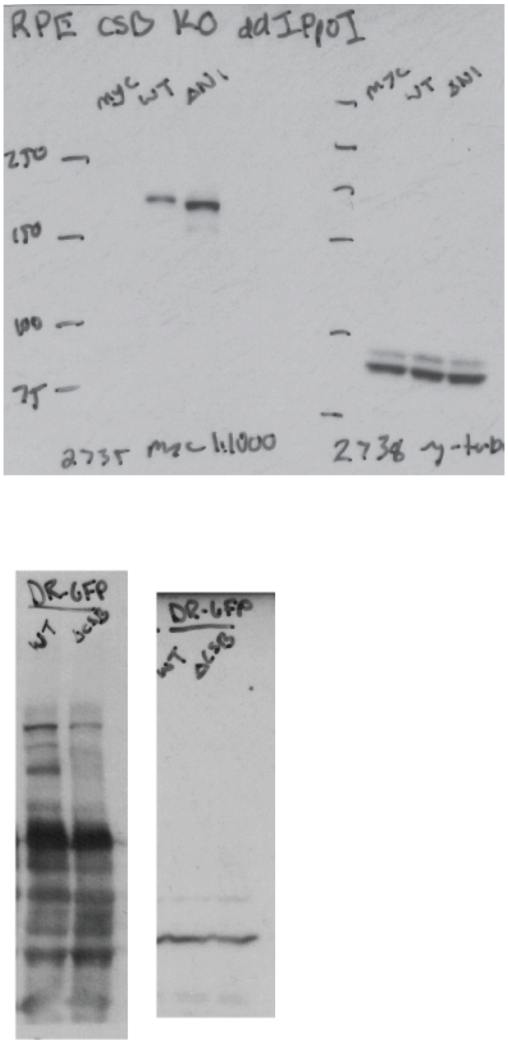

Supplementary Figure 10. Source data for western blots shown in Fig. 6c (top panel) and 6j (bottom panel).

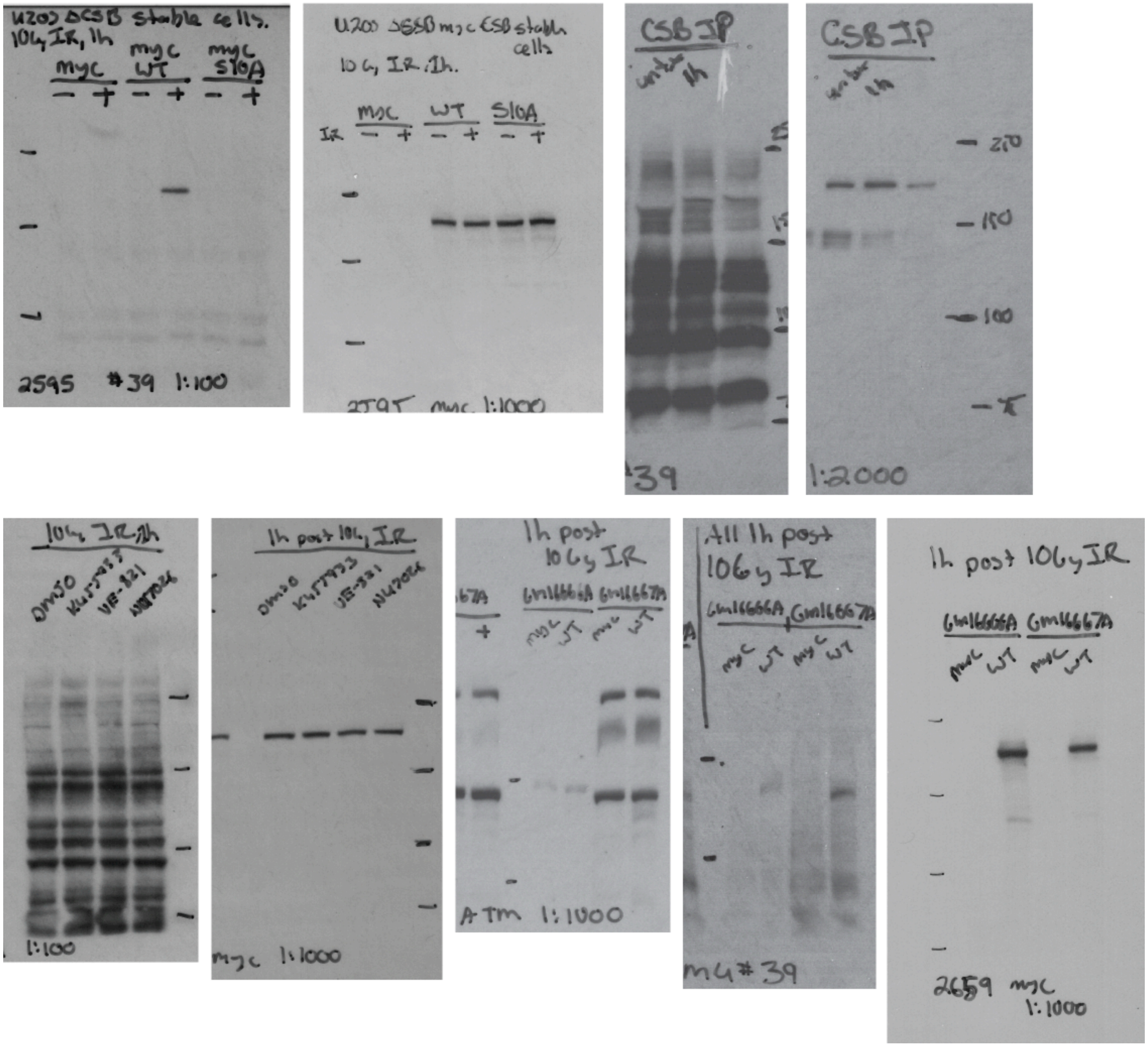

Supplementary Figure 10. Source data for western blots shown in Fig. 7a-7d.

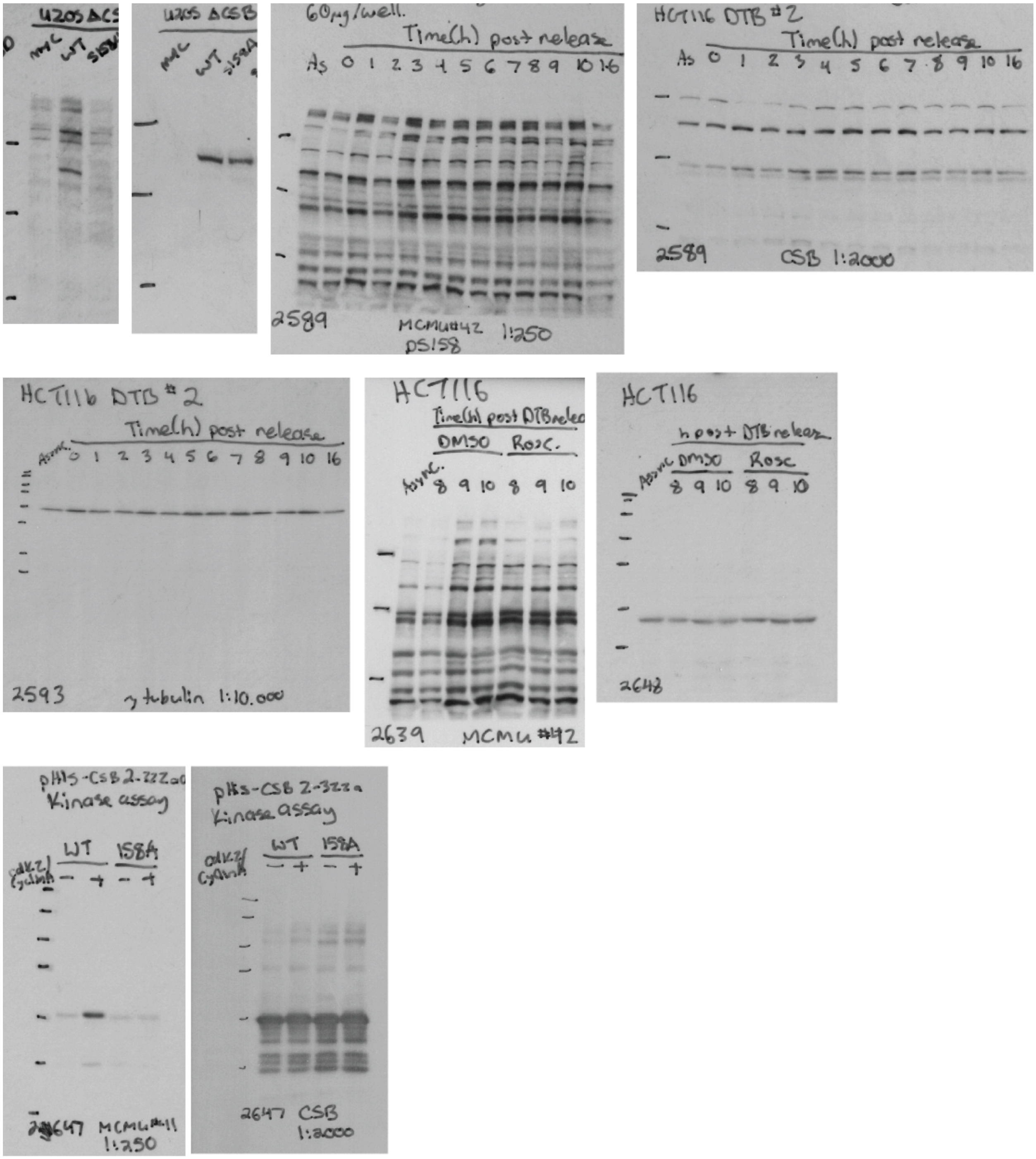

Supplementary Figure 10. Source data for western blots shown in Fig. 8a-8d.

Supplementary Table 1. Antibody List

Target	Mono/ polyclonal	Clone/ reference	Antibody raised in	Source	Western	IF	IP
53BP1	mono	612522	mouse	BD Sciences	1:2000	1:2000	
ATM	mono	2C1	Mouse	Novus Biologicals	1:500		
BRCA1	mono	MS110	mouse	Abcam			1μg
BRCA1	poly	07-434	rabbit	Millipore	1:2000	1:10000	
Cyclin A	mono	6E6	mouse	Abcam		1:100	
CSA/ERCC8	mono	sc376981	mouse	Santa Cruz	1:200		
CSB	mono	553C5a	mouse	Fitzgerald		1:500	1μg
CSB/ERCC6	poly	A301-354A	rabbit	Bethyl Laboratories	1:2000		
CSB-pS10	poly		rabbit	Xu-Dong Zhu	1:100		
CSB-pS158	poly		rabbit	Xu-Dong Zhu	1:250		
FK2	mono	04-263	mouse	Millipore		1:500	
H2A	poly	ab18255	rabbit	Abcam			1μg
H2B	poly	ab1790	rabbit	Abcam			1μg
MAD2L2	mono	sc135977	mouse	Santa Cruz		1:100	
mCherry	poly	NBP2-25157	rabbit	Novus Biologicals	1:10000		
anti-myc	mono	9E10	mouse	Calbiochem	1:1000	1:2000	1μg
Rad51	poly		rabbit	Jan Hoeijmakers		1:2000	
RIF1	poly	sc55979	goat	Santa Cruz	1:200	1:200	1μg
SMARCAD1	poly	A301-593A	rabbit	Bethyl Laboratories		1:500	
γH2AX	mono	05-636	mouse	Millipore		1:2000	
γ-tubulin	mono	GTU88	mouse	Sigma	1:20000		

Supplementary Table 2. Oligos used for PCR ChIP assays

Description		Primer Sequence	Size (bp)
Oligos for PCR ChIP assay, (I-PpoI cut site at 0 bp)			
	-8921	5'-GCAAGGGCTCATGAATGATAGTC-3'	263bp
		5'-CTTCCCCATTCAGAATTGTGATGAG-3'	
	-6245	5'-GCCTAAATGCCTCTTTCTACTGG-3'	236bp
		5'-GACACGGTTTTAGTGGAATGAGG-3'	
	-2930	5'-CTCTTAAACACTGGGTGCCTTTC-3'	252bp
		5'-CACAGCCAGTAAATGACAGAAATGG-3'	
	-496	5'-CTCCAGGGCATCCTTAGTGTT-3'	214bp
		5'-CAACGAGTATACTTGGGATGCG-3'	
	-281	5'- CTTTGCTGCTTTTTCTTCTTCTCC-3'	241bp
		5'- GACTTCTTTCCCACCAAGTCTTC-3'	
	408	5'-GTTCCCATTATCTGAAGAGCGTC-3'	238bp
		5'-CAAGGTCACTCAGCTTGTAAGG-3'	
	1756	5'-GGTTGTTCACACCCTTTCTGAG-3'	251bp
		5'-CCTGACTCACAGTAGACCCTC-3'	
	3559	5'-GCTTTGGCTTGTAACCCACAAC-3'	231bp
		5'-GAGTGTCTATCTACAGTGAGCCC-3'	
	5900	5'-CAGAGATGGAGGACAATTATGATGTG-3'	257bp
		5'-GCATGATCCTAAATTGTATGTACAGC-3'	
	7014	5'- GCATTCTGGAGTTCCTTGCTG -3'	233bp
		5'- GCACTTCCTTATCTCCACTCTTCC -3'	
	GAPDH site	5'-AAGCTTGTCATCAATGGAAATCCCATC-3'	548 bp
		5'-CTCAGACGGCAGGTCAGGTCCACCAC-3'	

Supplementary Table 3. Oligos used for real-time PCR ChIP assays

Description	Primer Sequence	Size (bp)
Oligos for real-time PCR ChIP assay (I-PpoI cut site at 0 bp)		
-6195	5'-TGACCTAAGGAACGAGCTAAACC-3'	126bp
	5'-GAGTAGGGGGAGTCCACAAGTC-3'	
-2907	5'-CAGTGGGTGGATTAACCTCTCTGA-3'	122bp
	5'-CCAACATCCATTTGTTAGTTCCCTT-3'	
-527	5'-CATGTATGTGGTCAGGACCTCC-3'	136bp
	5'-GAGAGAGAACTGACAATTGGGTTG-3'	
-168	5'-CCCAACTCCTTCACCAGCAAAT-3'	123bp
	5'-GGAGATGACTTCTTTCCCACCAAG-3'	
408	5'-GTTCCCATTATCTGAAGAGCGTC-3'	144bp
	5'-GTTGGATGGCTCTGATAGTTACAA -3'	
1756	5'-CACACCCTTTCTGAGTACACTGAGA -3'	122bp
	5'- GTCTTGTGACCTAATAGCGGAGAA-3'	
3559	5'-GCTTTGGCTTGTAACCCACAAC-3'	131bp
	5'-GATGCTGCTCATACCCAATGTA-3'	
7014	5'- GCATTCTGGAGTTCCTTGCTG -3'	95bp
	5'- CTAATGCACCCACTCATGCTTT -3'	
Oligos for real-time PCR DSB-induction assay		
I-PpoI site at chromosome 1 (flanking the I-PpoI site)	5'-CTTGGTGGGAAAGAAGTCATCTCC -3'	142bp
	5'-CTCTTTCCACTGTGGTATGAAACCT-3'	
GAPDH site	5'-GGCTTGCCCTGTCCAGTTAAT-3'	103bp
	5'-CTAGCTCAGCTGCACCCTTTA -3'	
